# Updating stored memory requires adult hippocampal neurogenesis

**DOI:** 10.1038/srep13993

**Published:** 2015-09-11

**Authors:** Irene Suárez-Pereira, Ángel M Carrión

**Affiliations:** 1 Departamento de Fisiología, Anatomía y Biología Celular, Universidad Pablo de Olavide, Carretera de Utrera Km 1, 41013 Sevilla, Spain

## Abstract

Adult hippocampal neurogenesis appears to influence hippocampal functions, such as memory formation for example. While adult hippocampal neurogenesis is known to be involved in hippocampal-dependent learning and consolidation processes, the role of such immature neurons in memory reconsolidation, a process involved in the modification of stored memories, remains unclear. Here, using a novel fast X-ray ablation protocol to deplete neurogenic cells, we have found that adult hippocampal neurogenesis is required to update object recognition stored memory more than to reinforce it. Indeed, we show that immature neurons were selectively recruited to hippocampal circuits during the updating of stored information. Thus, our data demonstrate a new role for neurogenesis in cognitive processes, adult hippocampal neurogenesis being required for the updating of stored OR memories. These findings suggest that manipulating adult neurogenesis may have a therapeutic application in conditions associated with traumatic stored memory, for example.

Following initial information acquisition, memory traces are committed to long-term memory through a consolidation process that stabilizes and stores the information acquired^1^. Consolidation of hippocampal-dependent memories, such as object recognition memory (ORM), requires new protein synthesis over a defined time course^2^,^3^. However, the efficiency of recovery of a consolidated ORM may show a time-dependent decrease^3^. Consolidated memories are not permanently fixed and they are malleable, susceptible to updating, strengthening or even loss due to further experiences^4^. Moreover, stabilization of a recalled memory relies on a further protein-dependent process called reconsolidation^5^.

Although consolidation and reconsolidation are processes that are conceptually similar and that share a dependence on protein synthesis, the brain regions and signalling pathways involved in these processes seem to differ^3^,^6^. Adult hippocampal neurogenesis (AHN) is involved in the consolidation of hippocampal-dependent memories^7^,^8^, yet the role of AHN in the reconsolidation of stored memories remains unclear. Here, using a protocol that rapidly ablates adult hippocampal precursors and immature neurons, we demonstrate that AHN is only required for object recognition memory reconsolidation when mice find novelty in a reactivation session. This notion is reinforced by the increase in the number of hippocampal immature neuron expressing Egr1, a molecular marker of neuron activation, after reactivation with novelty. Thus, these results associate AHN to the updating of stored memories.

## Results

**Figure 1:**
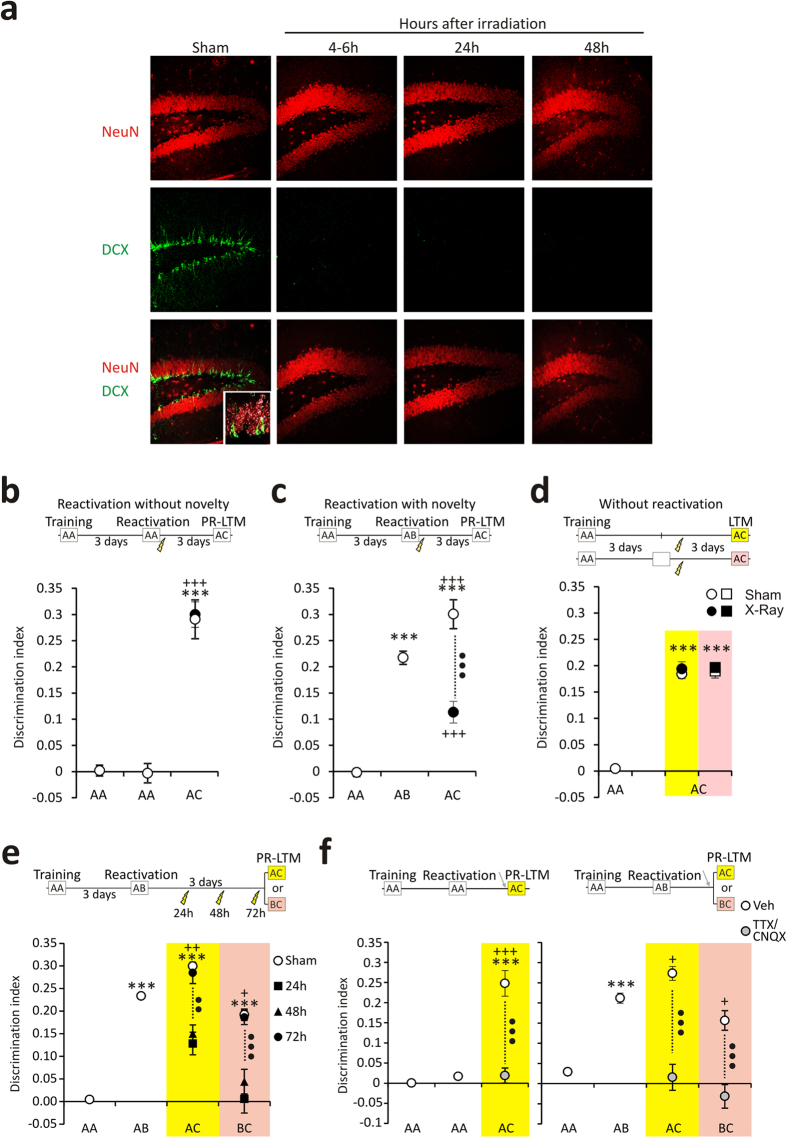
Adult hippocampal neurogenesis is required to update stored ORM. (**a**) Temporal effect of X-ray irradiation on the immature, doublecortin (DCX) labelled hippocampal cells. (**b**–**d**) The effect of depleting adult neurogenesis on reconsolidation. Reconsolidation in sham and irradiated mice was compared in three different circumstances: (**b**) reactivation without novelty; (**c**) reactivation with novelty; and (**d**) no reactivation with or without context exposure. In all cases irradiation was performed 3 days after the OR training. (**e**) Differential temporal requirements for the maturation of adult immature neurons in ORM reconsolidation. The temporal course of X-irradiation in the ORM studies is shown in the upper panel. (**f**) The functional role of the hippocampus in PR-LTM retrieval is confirmed by infusion of the TTX/CNQX cocktail (orange shadows). In each graph, the letters A, B and C represent the different objects used; *represent significant differences between the sessions and the training session in the same experimental group; ^+^represent significant differences between the LTM sessions and the reactivation session in the same experimental group; and • represent significant differences between the LTM session of each irradiated group with respect to the sham mice. A symbol, p < 0.05, two symbols, p < 0.01, and three symbols, p < 0.001.

To determine whether adult neurogenesis is required to carry out hippocampal-dependent reconsolidation, we have used a local irradiation method developed previously to rapidly eradicate neurogenesis in vigilant, movement restricted mice^8^, (Fig. 1a and Fig. S1a). The brain irradiation protocol does not produce collateral effects on mature neurons (Fig. S1b) nor a neuroinflammatory response (Fig. S2), yet it allows us to perform behavioural tests 4–6 hours after irradiation, a time window not accessible with standard methods.

To study object recognition (OR) reconsolidation processes, mice were left for 15 minutes in a small area containing two objects. Three days later, a 10 minute reactivation (RA) session was performed and after a further 3 days, a post-reactivation long-term memory (PR-LTM) test was performed as a measure of reconsolidation (Fig. 1b–d). In these experiments neurogenesis was ablated after the RA session. When familiar objects were used in the RA session, no differences in the discrimination index (DI) were seen between training and RA sessions (DI = 0.0 ± 0.01 and −0.01 ± 0.01 for the training and RA sessions, respectively). Also, when irradiation was performed after the RA session, the DI of irradiated mice was similar to that of sham mice in the PR-LTM (DI = 0.29 ± 0.04 for sham mice and 0.27 ± 0.03 for irradiated mice [t(14) = 0.54; p = 0.58]; Fig. 1b). By contrast, when RA was performed with a novel object and a familiar object irradiated mice displayed a lower DI in the PR-LTM test than the sham mice (DI = 0.29 ± 0.01 for sham mice and 0.11 ± 0.02 for irradiated mice [t(17) = 8.23; p < 0.001]; Fig. 1c). Finally, the effect of irradiation on reconsolidation required an object recognition RA session, since when the mice were only irradiated 3 days after the training session, or after a exploration session in the arena without objects, no change in the DI was evident in the PR-LTM test (DI = 0.18 ± 0.0 for sham mice and 0.19 ± 0.01 for irradiated mice [t(18) = 0.61; p = 0.54], and DI = 0.19 ± 0.01 and DI = 0.19 ± 0.00 for sham and irradiated exploration mice, respectively [t(11) = 0.46; p = 0.64]; Fig. 1d). Interestingly, when the same experiment described above was performed but using a different context for the object RA session, similar results were obtained (Fig. S3). All these results suggested that AHN was involved in reconsolidation only when novel objects were presented in the RA session (i.e., when the original OR memory had to be modified).

We wanted to assess the temporal relationship between adult neurogenesis and OR reconsolidation. To test this temporal association, OR reconsolidation was studied using the same protocol described above but irradiating mice 24, 48 or 72 hours after the novelty RA session. Mice that were irradiated 24 or 48 hours after the RA session displayed poor reconsolidation compared to non-irradiated mice (DI = 0.3 ± 0.01 for sham mice, and 0.12 ± 0.02 and 0.14 ± 0.02, for mice irradiated 24 and 48 h after RA, respectively [t(17) = 6.81 and 6.83 for comparisons of sham mice with mice irradiated 24 h after RA and with mice irradiated 48 h after RA, respectively; p < 0.001 in both cases]; Fig. 1e), whereas the reconsolidation index of mice irradiated 72 hours after RA was similar to that of non-irradiated mice [t(17) = 0.53; p = 0.62]. To assess whether reactivation with novelty also caused new information storage, a PR-LTM session was performed with the novel object used in the RA session (now a familiar object) and a new object. Using this protocol, sham mice showed preference for the new object. In order to test the temporal association between neurogenesis and OR reconsolidation in this new protocol, mice were irradiated 24, 48 or 72 hours after the novelty RA session. As in the reactivation protocol described above, mice that were irradiated 24 or 48 hours after the RA session displayed poor reconsolidation compared to non-irradiated mice, whereas the reconsolidation index of mice irradiated 72 hours after RA was similar to that of non-irradiated mice [DI = 0.19 ± 0.01, 0.0 ± 0.02, 0.04 ± 0.02 and 0.18 ± 0.01 for the sham mice, and for the mice irradiated for 24, 48 and 72 h after RA, respectively [t(14) = 5.82, 5.17 and 0.29 for comparisons between sham and mice irradiated 24 h, 48 h or 72 h after RA, respectively; p < 0.001 for the first two comparisons and p = 0.72 for the last comparison]. These results suggested that OR memory reactivation requires neurogenesis (or at least the presence of some of the different neurogenic states) for at least 3 days after the RA session.

However, we could not be sure that reactivation process was hippocampal-dependent 3 days after OR training. To resolve this issue, we blocked hippocampal activity by locally administering a TTX/CNQX cocktail prior to the PR-LTM test (Fig. 1f). TTX/CNQX diminished the reconsolidation indices in all cases, evidence that this process is indeed hippocampal-dependent. These results indicated that OR reconsolidation required immature neurons for at least 3 days after the RA session.

**Figure 2:**
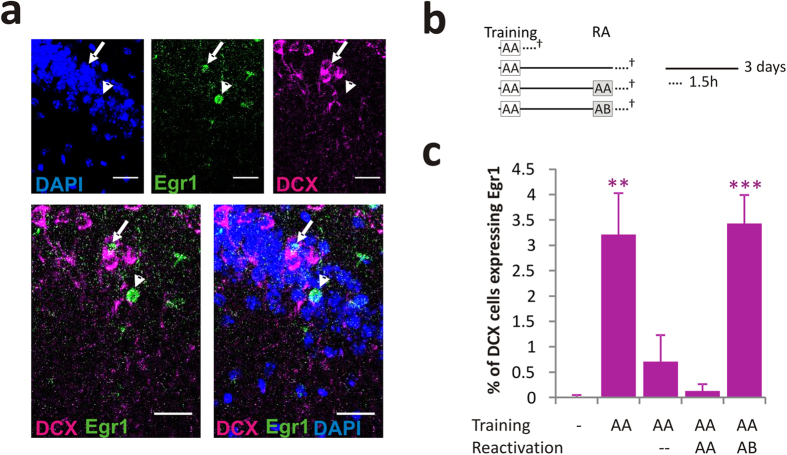
Stored memory retrieval with novelty recruits adult immature neurons to hippocampal circuits. (**a**) Representative microphotograph of immunofluorescence for Egr1 (green), doublecortin (DCX, purple) and DAPI (blue). Merged images of Egr1 and DCX, and of Egr1, DCX and DAPI are shown. The arrow and arrowhead indicate a DCX cell expressing Egr1 and granular neuron expressing Egr1, respectively. (**b**) Schematic representation of the experiment, where ^†^represents the sacrificing of the mice. (**c**) Quantification of DCX expressing cells labelled with Egr1 in the different experimental groups: *p < 0.05; **p < 0.01; and ***p < 0.001.

To obtain further evidence that AHN is involved in reconsolidation, we performed immunofluorescence studies to assess the distribution of Egr1, an immediate-early gene that is a marker of neuronal activity and circuit integration^9^ related to memory consolidation and reconsolidation^10^. Similarly, we evaluated the distribution of doublecortin (DCX), a prevalent marker of immature neurons in the dentate gyrus of non-trained, trained, non-reactivated and reactivated mice, with or without exposure to novelty (Fig. 2a,b). While little expression of Egr1 was detected in DCX^+^ cells in the unmanipulated (0 ± 0.5%), in the non-reactivated (0.71 ± 0.52%) and reactivated without novelty (0.13 ± 0.13%) mice, a significant increase in Egr1^+^/DCX^+^ cells was evident in mice sacrificed 1.5 h after training (3.21 ± 0.82%), as well as after the novelty RA session (3.43 ± 0.56%; Fig. 2c). These results suggest that hippocampal immature neurons are recruited to hippocampal circuits upon exposure to a novel feature in a familiar situation.

## Discussion

Long-term memory formation not only requires the stabilization of learned information but also, the subsequent capacity to modify the stored memory trace by further related experiences. These events occur through two related mechanisms, known as consolidation and reconsolidation, respectively. The first studies into the cellular/molecular mechanisms of consolidation and reconsolidation found these two processes to be similar^11^,^12^,^13^. However, both quantitative and qualitative differences in the gene and cellular processes associated with both phenomena have since been identified^14^,^15^. Moreover, functional pharmacological and gene knockdown studies have demonstrated that the consolidation and reconsolidation of a given memory are not dependent upon the same mechanisms^16^,^17^, even in the brain areas involved in both processes^3^,^18^,^19^.

With the discovery and acceptance of adult hippocampus neurogenesis (AHN), many studies carried out in the past decade have set out to establish the role of this phenomenon in learning and memory processes. In some such studies, neurogenesis was interrupted for long periods of time and conflicting results were obtained^7^. Such discrepancies may be due to differences in the ablation protocol or the cognitive paradigms used, or even in the alterations to compensatory neuronal circuits that result from the long-term inhibition of neurogenesis. However, all these studies focus more on adult generated mature neurons that on the different stages of the neurogenic process. In the past few years, manipulating neurogenesis over short periods of time has become a possibility^8^,^20^. We have developed an X-ray irradiation protocol^8^ that rapidly depletes the hippocampus of actively dividing neurogenic cells and around 90% of the DCX expressing cells, probably because these latter cells retain their capacity to divide albeit at a very low rate. In fact, DCX is expressed in hippocampal cells over a wide range of stages, from transiently amplifying progenitor cells to early post-mitotic cells^21^,^22^. Thus, the 10% of DCX expressing cells that survived irradiation might represent the more mature DCX expressing cells that can be distinguished from the rest of this population because they also express calretinin, a marker of immature post-mitotic cells during adult neurogenesis^23^. Using this fast X-ray depletion of adult immature cells, we found that neurogenesis is required for the acquisition and formation of long-term memory in object recognition and passive avoidance tests^8^. By contrast, selective inactivation of 4-week-old immature neurons by optogenetic techniques only provoked consolidation impairment in fear condition tests^20^. The differences between the results obtained may reflect the different periods of immature neuron ablation/inactivation.

Although neurogenesis is required for long-term memory formation, the possible role of immature cells in the reconsolidation process is unknown. The function of reconsolidation is to stabilize and reinforce a retrieved memory^24^,^25^ and/or to incorporate new information into the original stored memory trace^26^,^27^ depending on how the reactivation (RA) session is performed (with or without novelty). Here, and using an object recognition (OR) memory test, we demonstrate that AHN is required only when the reactivation session involves novelty and this process drives the updating of stored OR memories. Interestingly, the temporal requirement, three days, of neurogenesis after reactivation for reconsolidation is the same as that found for consolidation^8^. These temporal requirements suggest that the new born cell activated after training or after reactivation with novelty sessions mature into neurons in this period, and for this reason irradiation at this time does not affect memory. Interestingly, Egr1 is a transcription factor that is more strongly expressed in DCX positive cells under both circumstances, and it seems to be related with the activity-dependent selection and maturation of adult new born hippocampal cells^28^. Thus, these results indicate that both long-term OR memory formation and the updating of the stored object recognition memory have the same temporal requirement for neurogenesis.

Adult newborn granule cells generated from dividing dentate gyrus progenitors evolve through several stages to become mature granule cells that are indistinguishable from the granule cells born during embryonic development^29^,^30^,^31^. Indeed, these adult-generated neurons are known to be active in the formation and/or expression of memory^32^,^33^,^34^, indicating that they can integrate into functional hippocampal networks. However, whether the immature neurons generated in the adult hippocampus start to integrate into neuronal circuits before they mature completely is not known. We used Egr1 expression, a marker of neuronal activity and circuit integration^9^, combined with DCX, an immature neuron marker, to demonstrate that the activity of immature neurons changes after OR training and after RA with novelty. These results suggest that immature hippocampal DCX^+^ neurons may direct novel object recognition.

Taken together, our behavioural and histological results demonstrate that AHN is needed for reconsolidation processes only when it drives the updating of object recognition stored memories. If it were possible to deplete or interfere with this type of neurogenesis without producing any adverse cognitive effects, such an approach may be therapeutically useful to deal with certain conditions, such as post-traumatic stress disorder^35^.

## Materials and Methods

### Animals

The male Swiss (CD1) mice used in this study (5–6 weeks-old) were purchased from an authorized provider (University of Seville, Spain, or Janvier, France) and they were habituated to standard animals housing conditions for 2–3 weeks (a 12 h light/dark cycle, temperature and humidity). Behavioural studies were performed when the mice reached 8 weeks-of-age. All experiments were performed in accordance with European Union guidelines (2010/63/EU) and with Spanish regulations for the use of laboratory animals in chronic experiments (RD 53/2013 on the care of experimental animals: BOE 08/02/2013), and the approval of the University Pablo de Olavide animal care committees was obtained prior to performing this study.

### X-ray irradiation

The mice were immobilized in a plastic cylinder and positioned in the X-ray irradiation apparatus (MBR-1505R, HITACHI) as described elsewhere^8^. Animals were irradiated at 150 kV and 5 mA with a lead shield covering their entire body, except for the head. Radiation was administered for about 30 min at an approximate dose of 0.35 Gy/min and at a source-to-skin distance of 13 cm, delivering a total of 10 Gy. The slow rate at which irradiation is administered is probably responsible for allowing us to destroy the main part of the immature hippocampal cells by a mechanism that is not fully defined. Notably, this protocol did not affect the locomotion of the mice. Control animals were littermates handled similarly but not irradiated.

### Cannulation and Drug infusion

Mice were anesthetized with 4% chloral hydrate (10 μL/kg of body weight, i.p.) and when fully anesthetized, they were situated in a stereotactic frame. In order to inactivate the majority of the hippocampus, two pairs of stainless-steel guide cannulae were implanted bilaterally into the dorsal and ventral hippocampus of the mice at the following stereotactic coordinates: Dorsal, AP = −2.0 mm, ML = ±1.4 mm, V = −1.3 mm; and Ventral, AP = −3.2 mm, L = ±2.8 mm, V = −3.25 mm from the Bregma. The mice were then allowed to recover for at least 7 days. To transiently inactivate hippocampal activity, an inhibitory cocktail was prepared in PBS that contained tetrodotoxin (TTX [Tocris]: a sodium channel blocker, 20 μM) and 6-cyano-7-nitroquinoxaline (CNQX [Tocris]: a selective antagonist of AMPA receptors, 3 mM). The mice received infusions (0.5 μl) of the TTX-CNQX cocktail or PBS alone 30 minutes prior to the memory test, delivered through each cannula at a rate of 0.2 μl/min. The injection syringe (Hamilton) was left in place for 1 min following infusion.

### Object recognition (OR) memory

Mice were tested in a rectangular arena (55 × 40 × 40 cm) located in a room with dim lighting and constant background noise. The plastic objects used were of different shapes, colours and textures, and they were thoroughly cleansed with 70% ethanol between trials to ensure the absence of olfactory cues. The mice did not show any preference for any of the selected objects. The OR memory tests were performed as described in^3^. Briefly, two identical objects were placed in a rectangular arena during the training phase of the object recognition protocol. Subsequently, the animal’s memory of one of the original objects was assessed by comparing the amount of time spent exploring the novel object compared with that spent exploring the familiar one. The time spent exploring each object was recorded and the relative exploration of the novel object was expressed as a discrimination index [DI = (t_novel_ - t_familiar_)/(t_novel_ + t_familiar_)]. The criteria for exploration were based strictly on active exploration, circling or sitting on the object were not considered exploratory behaviours. Importantly, the exploration times in each session of the OR test were no different for the irradiated and sham mice (Table S1). All trials were performed by an experimenter blind to the drug treatments and/or manipulations, and each experimental group contained at least 7 mice.

### Experiment 1

The effect of depleting neurogenesis on OR memory reconsolidation was assessed three days after the end of the training session in a 10 minute reactivation session performed in a rectangular arena. Irradiation was administered after the end of a reactivation session, in which the mice were exposed to: 1, reactivation without novelty (with the same object used during training session); or 2, reactivation with novelty (with a familiar object used in training session and a novel object); or no object reactivation in the home cage or in the arena. Three days after this reactivation session finished, one object was changed for a novel one in order to test neurogenesis dependent post-reactivation long-term memory (PR-LTM).

### Experiment 2

To assess the temporal relationship between adult hippocampal neurogenesis and OR reconsolidation, mice were irradiated 24, 48 or 72 h after a reactivation session with novelty had terminated. PR-LTM was evaluated 3 days after reactivation with the object shown in Fig. 1e.

### Experiment 3

Hippocampal dependency of OR memory recall 3 days after reactivation. To evaluate the hippocampal dependency of our reconsolidation protocol, the hippocampus was inactivated by administering a TTX-CNQX cocktail 30 minutes before PR-LTM.

### Immunofluorescence and histological analysis

The brain of the mice was fixed by immersion in 4% paraformaldehyde in saline for 24 h at 4 °C. The tissue was cryoprotected in 30% sucrose-saline for 2 days at 4 °C, and then embedded in 30% sucrose and maintained at 4 °C. Immunofluorescence was performed on coronal brain sections (50 μm) essentially as described previously^36^. The sections were permeabilized with 1% Triton X-100 in PBS for 1 h, blocked in 5% (w/v) BSA, 1% Triton X-100 in PBS for 1h, and then incubated overnight at 4 °C with the primary antibodies of interest (see below) diluted in 1% (w/v) BSA, 1% Triton X-100 in PBS. The following day, the sections were rinsed for 1 h in PBS containing 0.1% Triton X-100, incubated for 1 h with the corresponding secondary antibodies diluted in 1% (w/v) BSA, 0.1% Triton X-100 in PBS, and after rinsing again with 0.1% Triton X-100 in PBS for 1 h and with PBS alone for 10 min, the sections were finally mounted in 50% glycerol. Primary antibodies against doublecortin (DCX, 1:100, Santa Cruz) and Egr1 (1:100 Santa Cruz) were used to visualize immature neurons and active cells, respectively. Primary antibody binding was visualized with an Alexa Fluor 488-conjugated anti-rabbit antibody or an Alexa Fluor 647-conjugated anti-goat antibody (1:500, Invitrogen) for Egr1 and DCX, respectively. The sections were counterstained with DAPI (1:5000) to visualise the cell nuclei. To minimize variability, at least 2 sections from the rostral (from −1.58 to −2.06 mm respect to Bregma) hippocampus were analysed per animal (n = 4 mice per experimental group) on a fluorescence confocal SPE DM 2500 microscope (Leica). In each section, the total number of labelled cells in the dentate gyrus area was quantified using Image-J software (downloaded as a free software package from the public domain: http://rsb.info.nih.gov/ij/download.html).

### Statistical analysis

The results were analyzed with the SPSS package for Windows and unless otherwise stated, the data represent the mean ± SEM values. The data were analyzed with one-way and two-way ANOVA, and a t-test was used for post-hoc comparisons.

## Additional Information

**How to cite this article**: Suárez-Pereira, I. and Carrión, Á M. Updating stored memory requires adult hippocampal neurogenesis. *Sci. Rep.* **5**, 13993; doi: 10.1038/srep13993 (2015).

## Supplementary Material

Supplementary Information
