# Supplementary material for: Updating stored memory requires adult hippocampal neurogenesis
Source: Sci Rep. 2015 Sep 11;5:13993. doi: 10.1038/srep13993 (PMC4566137; doi:10.1038/srep13993)
Supplement: Supplementary Information [file srep13993-s1.pdf]

Updating stored memory requires adult hippocampal neurogenesis

Irene Suárez-Pereira and Ángel M Carrión*

[1]Departamento de Fisiología, Anatomía y Biología Celular, Universidad Pablo de Olavide, Carretera de Utrera Km 1, 41013 Sevilla, Spain.

Inventory of Supplemental Information

Supplemental Figures. All Supplemental figures are related with Figure 1, and they show that our X-ray irradiation protocol does not affect to: mature neuron of dentate gyrus, and inflammatory processes.

Figure S1: X-ray irradiation impairs neurogenesis for at least 3 days. We show that irradiation protocol provoke a transient neurogenesis depletion without affect to mature neurons of dentate gyrus.

Figure S2: X-ray irradiation doesn't provoke a significant inflammatory response in the hippocampus.

Figure S3: Effect of the depletion of adult neurogenesis provoked by X-ray irradiation on object recognition memory reconsolidation when object recognition memory reactivation was performed in a novel context.

Supplemental tables.

Table S1 Object exploration time (seconds) per session in the ORM experiments. This table demonstrates that X-ray irradiation does not affect to mice exploratory activity. This table is related with Figure 1.

Table S2 Primer sequences used for RT-PCR gene expression analysis.

Supplemental Experimental Procedures

Immunohistochemistry, immunofluorescence and histological analysis showed in Supplementary figures S1 y S2.

RT-PCR gene expression. We perform a detailed description of gene expression experiments presented in Figure S2 of supplemental material.

Object recognition reconsolidation when reactivation and training sessions were performed in different contexts.

Supplemental Figures

Figure S1.

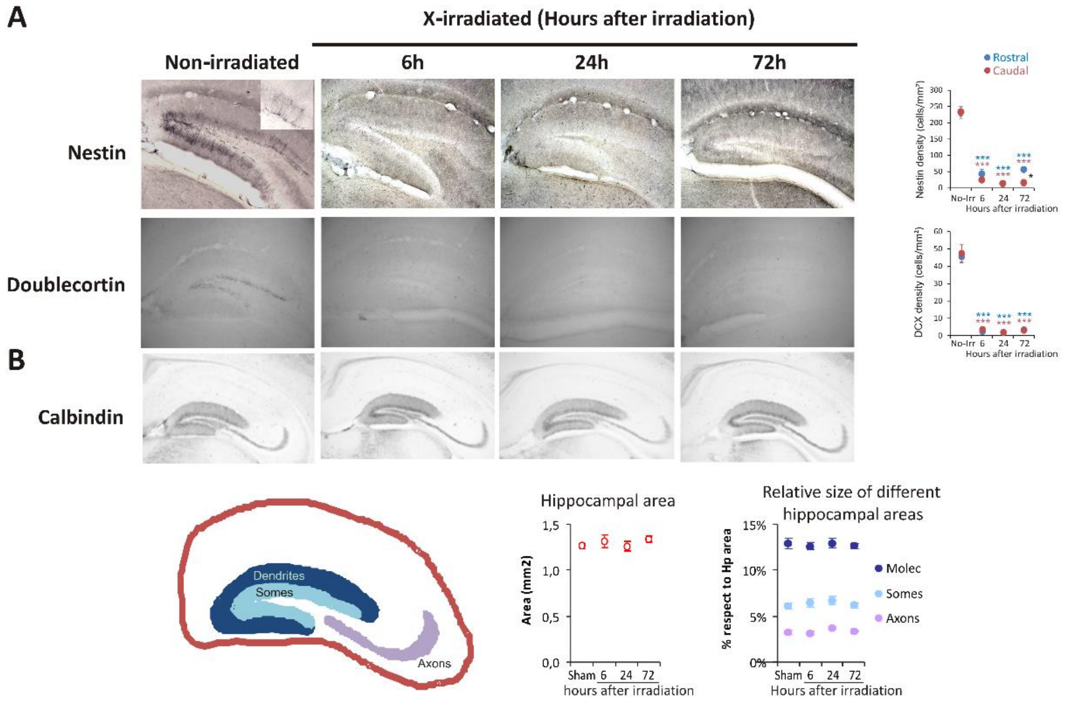

Supplemental Figure 1. X-ray irradiation impairs neurogenesis for at least 3 days. A, Representative microphotographs of immunohistochemistry for neural precusors (nestin), immature neurons (doublecortin, DCX) and mature neurons (calbindin) markers in the hippocampus of mice sacrificed 6, 24, and 72 hours after irradiation and in non-irradiated mice (sham). Around 85% of nestin cells and more than 90% DCX cells were lost in the first three days after irradiation. The inset in some microphotographs shows nestin positive cells at a higher magnification. The density of nestin and DCX expressing cells in the rostral (red plot) and caudal (blue plot) dentate gyrus of the hippocampus in mice sacrificed 6, 24, and 72 hours after irradiation, and in sham irradiated mice. **B,** Quantification of hippocampal surface and the relative area of the different part (dark blue, dendrites; light blue, somes; and lilac, axons) of calbindin positive neurons (granular cells) population in hippocampus of mice sacrificed 6, 24, and 72 hours after irradiation and in sham mice. No alterations in any of the parameter were detected. C, PCNA gene expression in the hippocampus of mice sacrificed 6, 24, and 72 hours after irradiation and in sham irradiated mice. A transient loss of PCNA is observed during the first 72 hours. n>5 mice per group.*, p<0.05; **, p<0.01; and ***, p<0.001.

Figure S2.

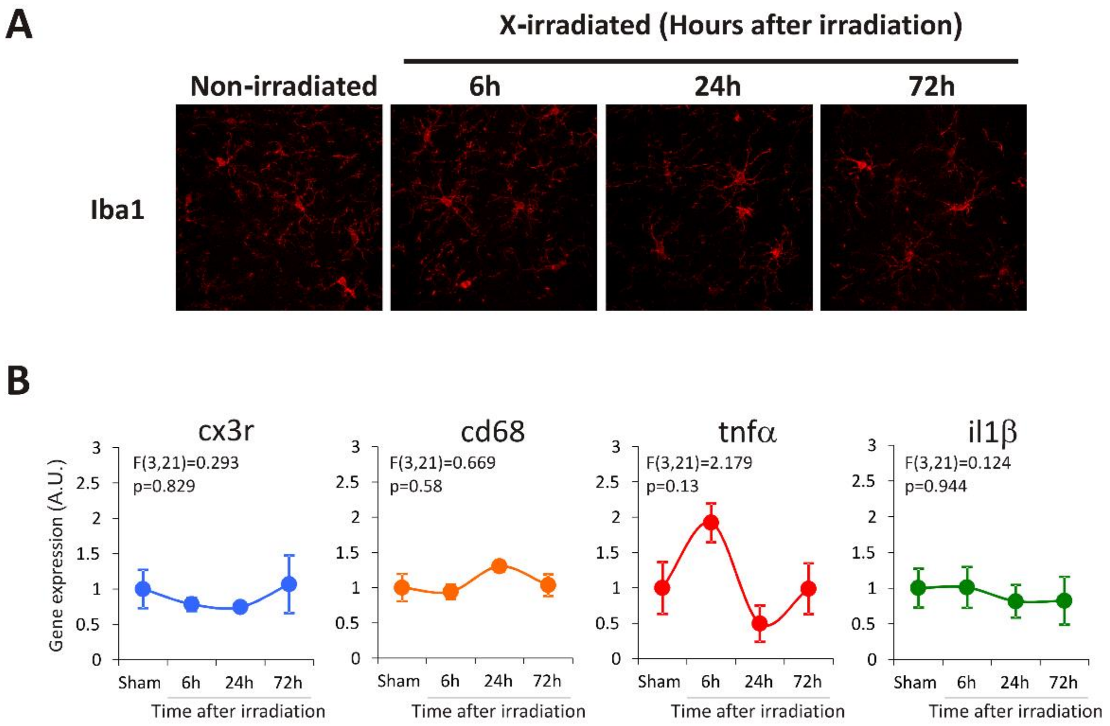

Supplemental Figure 2. X-ray irradiation does not provoke significant inflammatory response in the hippocampus. A, Representative immunofluorescence microphotographs for the microglial marker Iba1 in the hippocampus of mice sacrificed 6, 24 and 72 hours after irradiation, and in non-irradiated (sham) mice. The number and morphology of microglial cells did not change after X-ray irradiation. **B,** Gene expression analysis of microglial marker activation (cd68, cx3cr, tnfα, and illβ) in the hippocampus of mice sacrificed 6, 24 and 72 hours after irradiation, and in sham mice. Only a non-significant weak and transient increase in tnfα gene expression is observed. n>5 mice for sham groups and 4-5 mice for each of the irradiated groups.

Figure S3

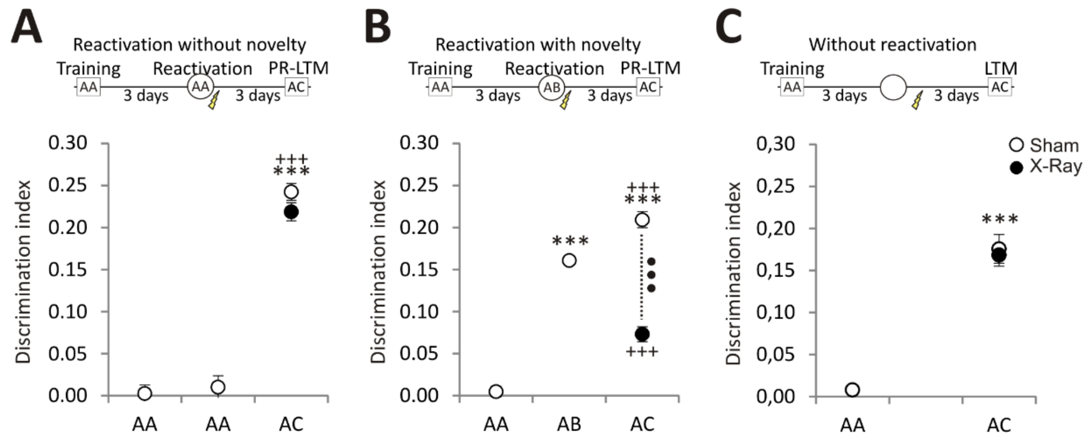

Supplemental Figure 3. Effect of adult neurogenesis depletion on object recognition memory reconsolidation when reactivation is performed in a novel context. Reconsolidation in sham and irradiated mice was compared in three different circumstances: **A,** reactivation without novelty; **B,** reactivation with novelty; and **C,** no reactivation. In all cases, irradiation was performed 3 days after OR training and after reactivation in a context different (circle) to that used in the training session (rectangle). In each graph, the letters A, B and C represent the different objects used: * represent significant differences between the sessions and with the training session in the same experimental group; + represent significant differences between the LTM sessions and the reactivation session in the same experimental group; and ● represent significant differences between the LTM session of each irradiated group with respect to the sham mice (one symbol, $p<0.05$, two symbols, $p<0.01$ and three symbols, $p<0.001$).

Supplemental tables.

Table S1. Object exploration time (seconds) per session in the ORM experiments.

	Sessions				
**Experiment**	**Training**	**Reactivation (RA)**	**LTM (A/C exploration times)**		**n**
**Figure 1. Sensibility of reactivation to adult immature neuron depletion**					
**b. Reactivation without novelty**					
**Sham**	118±4.08	61.2±8.52	82.4±4.73 (29±1.81/53.4±3.98)		8
**X-Ray 4h after RA session**	115.2±7.73	64±6.14	72.2±3.21 (27.2±1.98/45±1.51)		7
**c. Reactivation with novelty**					
**Sham**	112.54±5.23	70.63±4.94	64.36±4.53 (22.87±1.7/41.54±2.9)		10
**X-Ray 4h after RA session**	117.4±9.41	68±5.68	61.28±3.99 (27±1.63/34.28±2.43)		8
**d. Without reactivation session**					
**Sham**	106.83±7.59	--	56.16±5.23 (23±2.3/33.16±3)		12
**X-Ray 3 days after RA session**	128.85±7.75	--	54,2±7.59 (20.85±2.45/31.42±3.22)		7
**Sham arena exploration session**	129.33±4.01	--	50.16±3.17 (20±1.43/30.16±1.85)		6
**X-Ray 4h after arena exploration session**	125.5±4.25	--	40.33±2.07 (16±0.57/24.53±1.52)		6
**Figure 1e. Adult immature neuron temporal requirement for post-reactivation LTM formation**					
**Objects**	**AA**	**AB**	**AC**	**BC**	**n**
**Sham**	106±11.71	64.6±3.4	56.2±3.68 (19.8±1.39/36.4±2.71)	64±5.24 (25±3.53/39±2.23)	10
**X-Ray 24h after RA**	112±3.56	76±2.3	61.83±4.57 (26.83±1.81/35±2.82)	81.8±6.56 (41±3.06/40.8±3.77)	8
**X-Ray 48h after RA**	115.09±3.25	70.45±3.69	57.5±2.73 (24.66±2.15/32.83±1.62)	77.8±6.39 (37±2.7/40.8±3.87)	8
**X-Ray 72h after RA**	109.6±4.38	70.9±3.87	59.8±4.85 (21.4±1.63/36.4±2.71)	67±8.05 (26.8±3.26/40.2±4.82)	8
**Figure 1f. Hippocampal-dependent reconsolidation 72h after reactivation**					
**Groups**	**Training**	**Reactivation (RA)**	**LTM (A/C exploration times)**		**n**
**Sham RA without**	114±5.77	67.66±5.33	85±6.92 (32±1.73/53±5.19)		7

**(w/o) novelty**				
**TTX/CNQX RA w/o novelty**	112.4±5.12	62.6±5.71	69.4±4.1 (34±2.34/35.4±1.88)	7
**Sham RA with (w) novelty (AC)**	116.33±11.02	89±14.73	69±11.71 (25.66±3.52/43.33±8.51)	7
**TTX/CNQX RA w novelty (AC)**	111.8±5.56	92.4±9.22	89.6±6.2 (43.4±2.61/46.2±3.72)	7
**Sham RA with novelty (BC)**	128±9.23	96±13.79	102±9.64 (41.33±4.48/60.66±5.2)	7
**TTX/CNQX RA w novelty (BC)**	118.8±12.21	100±4.43	98.2±6.91 (50.8±4.35/47.4±2.61)	7

Table S2. Primer sequences used for RT-PCR gene expression analysis

gapdh-F	5´- GTAGGCCAAGTTGCCTTGTCCGT -3´
gapdh-R	5´- ATGTTCCAGTATGACTCCACTCACG -3´
cd68-F	5´- GGGGCTCTTGGGAACTACAC-3´
cd68-R	5´- GTACCGTCACAACCTCCCTG-3´
cx3cr-F	5´- GCCTCTGGTGGAGTCTGCGTG-3´
cx3cr-R	5´- TGGGCTTCCGGCTGTTGGTG-3´
tnfα-F	5´- GGCAGGTCTACTTTGGGAGTCATTGC-3´
tnfα-R	5´- ACATTCGAGGCTCCAGTGAATTCGG-3´
il1β-F	5´- AAAAGCCTCGTGCTGTCGGAC-3´
il1β-R	5´- GCAGGGTGGGTGTGCCGTCT-3´

Supplemental Experimental Procedures

Immunohistochemistry, immunofluorescence and histological analysis

For immunohistochemistry (IHC), antibodies against calbindin (1:3,000, Swant), nestin (1:250, Genetex) and doublecortin (dcx, 1:500, Santa Cruz) were used. For immunofluorescence (IF), an antibody against Iba1 (1:500, Wako) was used. Antibody staining for IHC was visualized with H2O2 and diaminobenzidine, and for IF with an Alexa Fluor 594-conjugated donkey anti-rabbit antibody (1:500, Invitrogen). To minimize variability, at least 2 sections from the rostral (from -1.58 to -2.06mm respect to Bregma) and caudal (from -2.92 to -3.50mm to Bregma) hippocampus were analyzed per animal under a bright-field DMRB RFY HC microscope (Leica) for IHC or a fluorescence confocal SPE DM 2500 microscope (Leica) for IF. In each section, the total number of positive cells and the dentate gyrus area was quantified using Image-J software (downloaded as a free software package from the public domain: http://rsb.info.nih.gov/ij/download.html).

Reverse transcription-PCR analysis of mRNA

Total RNA was extracted using the Tripure reagent (Roche Products) from the brain tissue of at least of six animals per group, collected from at least two different experimental sessions. More detailed information on the primers used can be found in Table S2. The values obtained were normalized with respect glyceraldehyde-3-phosphate dehydrogenase (GAPDH) expression and they are expressed relative to those of the non-irradiated animals to which a value of 1 was assigned.

Object recognition reconsolidation when reactivation and training sessions were performed in different contexts

The effect of depleted neurogenesis on OR memory reconsolidation was assessed using a 10 minute reactivation session performed in a circular arena (new context) three days after the end of the training session performed in a rectangular arena. Irradiation was administered 4h after the end of a reactivation session, in which the mice were exposed to: 1, reactivation without novelty (the same object as that used during the training session); or 2, reactivation with novelty (with a familiar object used in the training session and then a novel object); or no object reactivation in the circular arena. Three days after this reactivation session finished, one object in the rectangular arena was changed for a novel one in order to test neurogenesis dependent post-reactivation of long-term memory (PR-LTM).
